# Molecular Hydrogen and Extracorporeal Gas Exchange: A Match Made in Heaven? An In Vitro Pilot Study

**DOI:** 10.3390/biomedicines12081883

**Published:** 2024-08-18

**Authors:** Foivos Leonidas Mouzakis, Flutura Hima, Ali Kashefi, Johannes Greven, Lothar Rink, Emiel P. C. van der Vorst, Joachim Jankowski, Khosrow Mottaghy, Jan Spillner

**Affiliations:** 1 ECC Lab, Institute of Physiology, Medical Faculty, RWTH Aachen University, 52074 Aachen, Germany; 2 Department of Thoracic Surgery, Medical Faculty, RWTH Aachen University, 52074 Aachen, Germany; 3 Institute of Immunology, Medical Faculty, RWTH Aachen University, 52074 Aachen, Germany; 4 Institute for Molecular Cardiovascular Research, Medical Faculty, RWTH Aachen University, 52074 Aachen, Germany; 5 Aachen-Maastricht Institute for CardioRenal Disease (AMICARE), RWTH Aachen University, 52074 Aachen, Germany; 6 Interdisciplinary Center for Clinical Research (IZKF), RWTH Aachen University, 52074 Aachen, Germany; 7 Institute for Cardiovascular Prevention (IPEK), Ludwig-Maximilians-Universität, 80336 München, Germany; 8 Department of Pathology, Cardiovascular Research Institute Maastricht (CARIM), University of Maastricht, 6200 MD Maastricht, The Netherlands

**Keywords:** extracorporeal circulation, molecular hydrogen, ECMO, oxidative stress, inflammation, anti-inflammatory action

## Abstract

Extracorporeal circulation (ECC) is frequently implemented in a vast array of modalities such as hemodialysis, cardiopulmonary bypass, extracorporeal membrane oxygenation (ECMO), and others. Patients receiving any such therapy are frequently encumbered with chronic inflammation, which is inherently accompanied by oxidative stress. However, ECC treatments themselves are also responsible for sustaining or promoting inflammation. On these grounds, an in vitro study was designed to investigate the therapeutic potential of molecular hydrogen (H_2_) against pro-inflammatory agents in ECC settings. Five miniature ECMO circuits and a small vial (Control) were primed with heparinized blood from healthy adult donors (*n* = 7). Three of the ECMO systems were injected with lipopolysaccharide (LPS), out of which one was additionally treated with an H_2_ gas mixture. After 6 h, samples were drawn for the assessment of specific biomarkers (MCP-1, MPO, MDA-a, TRX1, and IL-6). Preliminary results indicate a progressive oxidative and inflammatory response between the six systems. Circulation has triggered inflammation and blood trauma, but the staggering influence of LPS in this outcome is indisputable. Accordingly, hydrogen’s remedial potential becomes immediately apparent as biomarker concentrations tend to be lower in the H_2_-handled circuit. Future research should have distinct objectives (e.g., dosage/duration/cycle of hydrogen administration) in order to ascertain the optimal protocol for patient treatment.

## 1. Introduction

The term artificial organs pertains to devices and therapies capable of reproducing the function of human organs either partially or fully. Often, these constitute long-term, implantable solutions, whereas on other occasions, they can only be implemented for a certain period or in intermittent sessions. Apparatus and treatments for the lung, kidney, and liver fall into the last category, which necessitates the extracorporeal handling of blood. Patients suffering from impaired function of any of the aforementioned organs are also frequently afflicted by topical or systemic inflammation and/or oxidative stress, regularly exacerbated by the extracorporeal treatment [1,2,3,4].

Recently, the outbreak of the COVID-19 pandemic skyrocketed the utilization of extracorporeal membrane oxygenation (ECMO), rendering the study of oxidative/inflammatory phenomena in treated patients extremely pertinent [5,6]. According to Millar et al., the application of ECMO in critically ill patients is associated with a systemic inflammatory response, which can lead to organ injury and dysfunction [7]. This reaction is characterized by a rapid increase in plasma concentrations of inflammatory cytokines, such as TNF-α and IL-8, during the first 2 h of ECMO, potentially due to the release of pre-formed stores in the intestine [8]. The inflammatory response is further exacerbated by the exposure of a patient’s blood to the non-endothelialized surface of the ECMO circuit, which activates the innate immune system and can lead to inflammation and organ injury [3,4,7].

Studies have established that hydrogen gas possesses anti-inflammatory properties and can improve survival rates and organ damage in models of generalized inflammation [9,10]. This is particularly relevant in the context of ECMO treatment, which can lead to an excessive systemic inflammatory response [3,11]. The potential of hydrogen gas as a therapeutic agent in emergency and critical care medicine has already been demonstrated by Sano et al. in 2017 [12], whereas its continued application as an antioxidant in hemodialysis dates from 2009 [13,14]. Furthermore, it has been recommended for use in the treatment of various systemic diseases due to its anti-inflammatory and antioxidant abilities [15,16]. In the context of lung injury, a 2% supplement of hydrogen gas during mechanical ventilation (MV) support effectuated a reduction in the ventilator-induced inflammatory response and lower levels of epithelial apoptosis after 2 h of treatment [17,18]. Likewise, rats treated with hydrogen while on ECMO and MV exhibited 250% higher survival rates compared to the placebo group 4 h after resuscitation from cardiac arrest [19]. By the happenstance of COVID-19 hydrogen had already gained substantial momentum as an antioxidant and anti-inflammatory agent, hence the numerous postulations of its efficacy against the side effects of the viral infection [15,20,21,22].

These findings suggest that hydrogen gas may play a vital role in mitigating inflammation in ECC settings. In furtherance of validating this thesis, an in vitro study was designed with clinical relevance in mind. Emphasis has been placed on investigating hydrogen’s impact during gas exchange, as the second most frequently encountered extracorporeal circulation modality after hemodialysis [23]. Blood from healthy human donors circulated in small ECMO circuits in order to observe the influence of diverse parameters (circuit components, introduction of pathogens, and hydrogen treatment) on the provocation of an inflammatory response. The preliminary results communicated here corroborate the feasibility of this study and pave the way for prospective exhaustive in vitro trials, where the specific characteristics of hydrogen-enriched ECMO treatments ought to be further investigated and explicitly determined ahead of any in vivo applications.

## 2. Materials and Methods

### 2.1. Donors

On account of being viewed as a pilot study since its inception, and in pursuance of minimizing the margin of error, a few straightforward criteria were set for the selection of blood donors:

- should be healthy adults
- should be capable of donating > 300 mL of blood without complications
- should have an average hematocrit Hct ≥ 42%
- should not have experienced acute pyretic or other inflammatory incidents recently
- should not have any blood-associated conditions
- should not have a prescription for anticoagulation medication, etc.

Based on the above, female donors were excluded during this preliminary phase. The study was conducted in accordance with the Declaration of Helsinki and was approved by the RWTH Aachen Medical Faculty Institutional Ethics Committee (EK 23–234, extended approval from 20 July 2023).

### 2.2. In Vitro Inflammation

As per the selection criteria stipulated above, the donated blood should be free from any inflammation and oxidation prior to any further handling. Yet, for the purposes of this study, an inflammatory response needed to be artificially triggered on demand. A solution was offered in the form of endotoxins, which promote the secretion of pro-inflammatory cytokines [24]. To determine the optimal dosage, a titrated solution of Lipopolysaccharide (LPS) was introduced in samples of donated blood. After 6 hours, plasma from each sample was implemented in the measurement of Interleukin 6 (IL-6) concentration via an enzyme-linked immunosorbent serologic assay (ELISA).

### 2.3. Experimental Setup

In pursuance of examining every alternative possible, six systems (5× ECMO, 1× Control), each serving a different purpose, were tested simultaneously in vitro. Three circuits were infused with 250 ng mL^−1^ of LPS each in order to artificially set off an inflammatory response, whereas the rest remained uncontaminated. The latter would contribute to the evaluation of the pro-inflammatory influence of extracorporeal circulation itself.

To facilitate the delivery of hydrogen gas (Hydrogen 5.0, Linde AG, Munich, Germany), a gas exchanger module was annexed to one of the LPS-infused systems. The supplied gas mixture contained roughly 6% H_2_, resulting in a dissolved H_2_ concentration of 100–120 ppb. Two more circuits, one of which was also LPS-treated, were also furnished with identical gas exchangers to keep track of any ramifications stemming from the gas exchange and from the integration of such modules. Naturally, the gas admixture that these two systems received was devoid of hydrogen and aimed to maintain blood gas values within the physiological range. The implemented gas exchanger modules had a surface area, A_eff_, of 0.3 m^2^ and 20 mL of priming volume.

**Figure 1:**
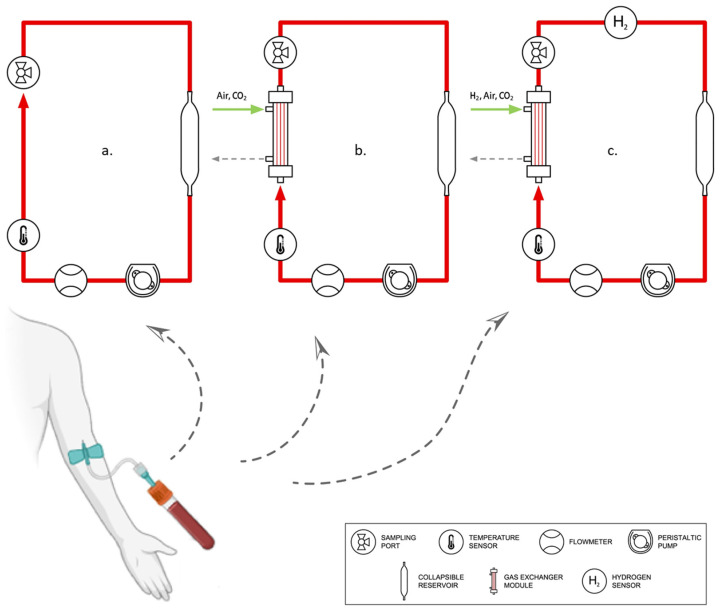
Graphic illustration of the circuit groups used in this study: a. Ref/LPS, b. Ref*/LPS*, c. LPS*H_2_.

All five ECMO circuits comprised a peristaltic pump (Stoeckert Instrumente GmbH, Munich, Germany), 3/16” and 1/4” tubing, and a collapsible reservoir made out of thin silicone. The control setup, consisting of a reaction tube filled with untreated blood, was left standing for the entire duration of the experiment (t_exp_ = 6 h). Table 1 summarizes the essential characteristics of each setup and their corresponding labeling for disambiguation, whereas Figure 1 portrays the predominant variations.

### 2.4. Test Protocol

Upon obtaining informed consent and explaining the nature and potential consequences of the study, venous blood (ca. 300 mL) was drawn from healthy male donors with a median age of 37 (28, 55). Sodium heparin (B. Braun, Melsungen, Germany) was instilled directly in the blood containers as an anticoagulant (10 IU mL^−1^). In total, seven experiments (*n* = 7) were carried out. On each occasion, all six systems were primed with blood from a single donor. 

Once primed, the ECMO circuits were perfused at a blood flow rate of 40 mL min^−1^ for 6 hours uninterrupted. Blood samples (200 μL) were drawn from each circuit at regular intervals to determine the partial pressures of oxygen (pO_2_) and carbon dioxide (pCO_2_), hemoglobin (Hb) levels, and other clinically relevant blood gas data. A blood gas analyzer (Radiometer, Copenhagen, Denmark) facilitated this procedure. Base excess was the only parameter adjusted, as per ISO 7199:2016 [25], to prevent any acidosis/alkalosis phenomena that could induce further oxidative stress and/or blood trauma.

Blood dilution was avoided to retain the maximum number of cells possible (mean hemoglobin Hb = 14 g dL^−1^ ± 0.7). Blood flow rate and pressure drop were monitored by an ultrasonic flowmeter (Transonic, Ithaka, NY, USA) and a pressure gauge (CODAN pvb Critical Care Inc, Forstinning, Germany), respectively, whereas gas supply was regulated via gas flowmeters (Platon, Vienna, Austria). A contactless hydrogen sensor (Pureron Co. Ltd., Iwaki, Japan) registered the concentration of dissolved hydrogen in the LPS*H_2_ circuit, as Figure 1 attests.

At the end of each experiment, blood samples were taken from each circuit for further analysis, in volumes sufficient (4–5 mL) for multiple measurements. Plasma, extracted through centrifugation (14000× *g*, 5 min, RT), was used for the estimation of oxidative and inflammatory stress by measuring the concentrations of specific biomarkers (dual measurement). Likewise, plasma was implemented in the determination of blood trauma via spectrophotometry (Pharmacia Biotech, Uppsala, Sweden).

An experimental runtime of 6 h offered ample time both for protein expression and for hydrogen to react with any reactive oxygen species (ROS) in the system. On the other hand, it prevented any compromise of erythrocyte integrity by paying heed to blood damage evaluation protocols [26]. 

For standardization purposes, particularly since priming volume differed significantly between circuits, the Normalized Index of Hemolysis (NIH) was used for the estimation of blood trauma, as expressed by Equation (1) [27].

(1) $$NIH=\Delta PfHb\cdot V_{prim}\cdot \frac{100-Hct}{100}\cdot \frac{100}{Q_{B}\cdot t_{exp}}$$

### 2.5. Biomarkers of Inflammation and Oxidative Stress

Since the scope of this study was not as broad as the actual spectrum of biomarkers associated with oxidative and inflammatory responses, a few select samples were singled out on account of their frequent emergence in the relevant literature [28,29,30,31,32,33,34,35,36]. An essential parameter was the inclusion of representative biomarkers from both pools (oxidative and inflammatory), as Table 2 signifies. Biomarker assessment was carried out by means of ELISA, performed as per the manufacturer’s protocol (Thermo Fisher Scientific, Waltham, MA, USA).

### 2.6. Statistics

Statistical analysis (multivariate analysis of variance—ANOVA) was conducted to inspect the level of significance with *p* < 0.05 using the software GraphPad Prism version 9.1.1 (GraphPad Software, Inc., San Diego, CA, USA).

## 3. Results

**Figure 2:**
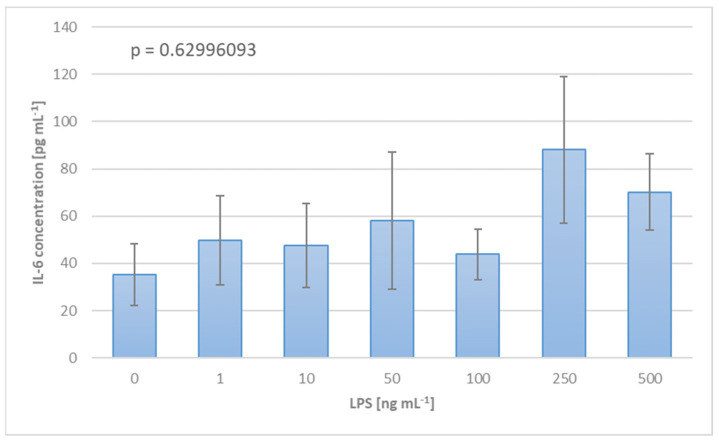
Method for establishing the optimal LPS dosage for the experimental protocol based on the inflammatory reaction of blood to titrated LPS solution, expressed in terms of IL-6 concentration.

Determining the optimal LPS dosage for the in vitro investigations was crucial for the quality and accuracy of the experimental outcome. During a pre-trial, blood samples were subjected to titrated LPS solution in order to trigger an inflammatory response. The results, presented in Figure 2, point to an LPS concentration of 250 ng mL^−1^, for which the maximum IL-6 expression can be observed.

**Figure 3:**
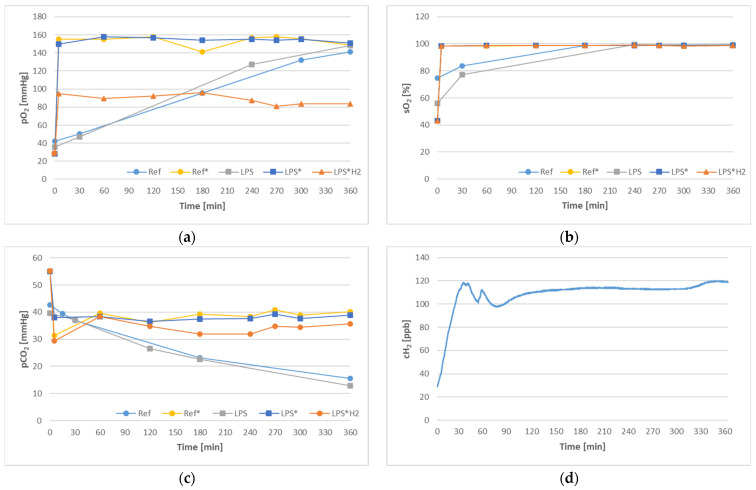
Gas exchange in each circuit over the course of the investigation, in terms of oxygen partial pressure (**a**) and saturation (**b**), carbon dioxide partial pressure (**c**), and hydrogen concentration (**d**).

Figure 3 displays individual blood gas parameters, as well as aspects of gas exchange for each of the tested circuits. The partial pressure of oxygen and the corresponding saturation are depicted in Figure 3a,b, revealing the importance of the gas exchanger module in achieving a steady state early on. The Ref and LPS circuits, in contrast, were only subject to passive gas exchange through the tubing walls, hence the delayed accomplishment of the final values. Likewise, carbon dioxide rapidly reached equilibrium values in the Ref*, LPS*, and LPS*H_2_ circuits, as portrayed in terms of partial pressure in Figure 3c, while the non-aerated circuits exhibited gradual losses through the conduit. Hydrogen transfer is represented in Figure 3d in the form of the concentration of molecular hydrogen dissolved in the circulating blood. The rendered charts depict average values, while standard deviations were insignificant and shall therefore not be annotated.

**Figure 4:**
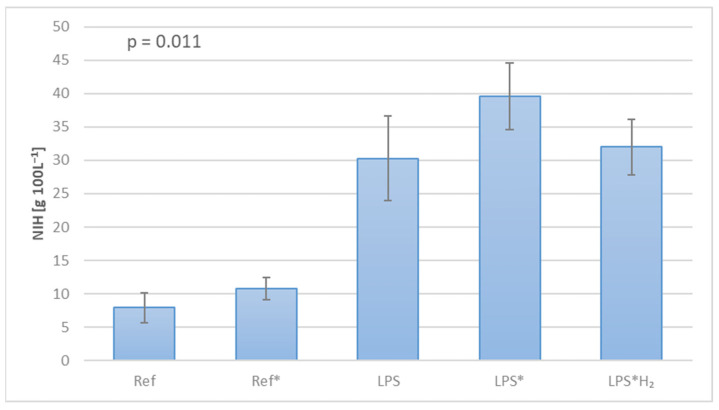
Normalized Index of Hemolysis for each circuit, as a sign of blood trauma.

In furtherance of assessing the influence of diverse factors (i.e., circuit design, component selection, and test protocol) on experimental conduct, the hemolysis rate was measured and the Normalized Index of Hemolysis was calculated for each circuit. Moreover, this process effectuated the individual estimation of mechanically vs. biochemically induced hemolysis, providing valuable insight into the kinetics of these investigations. Figure 4 discloses evidence of pronounced blood trauma in the systems, where an inflammatory reaction was artificially triggered, whereas an unprecedented amelioration manifests in the circuit handled with H_2_.

**Figure 5:**
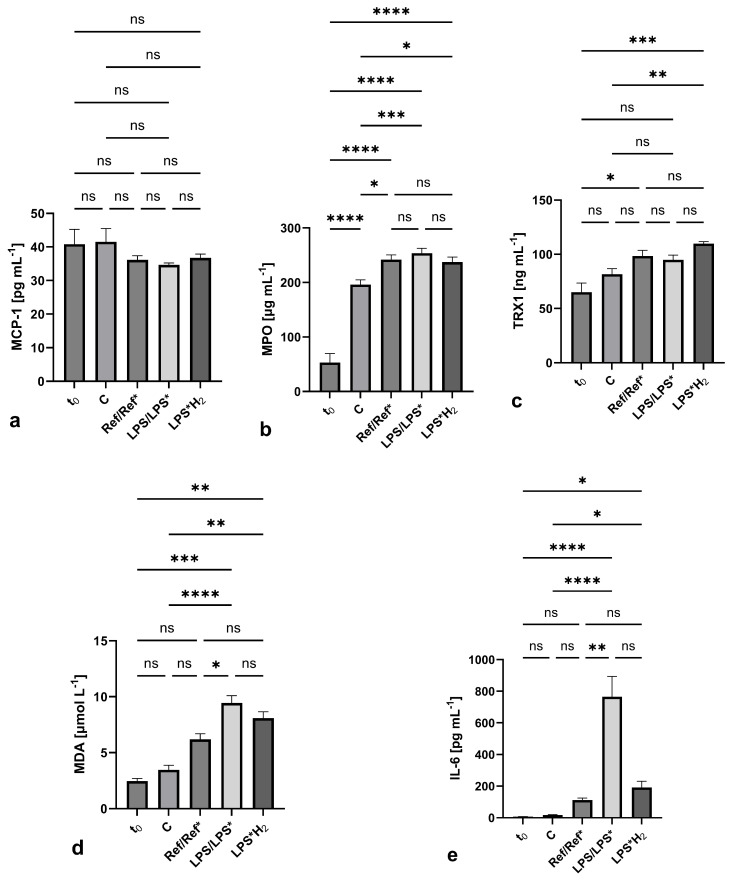
Biomarker expression in every circuit: (**a**) MCP-1, (**b**) MPO, (**c**) TRX1, (**d**) MDA, and (**e**) IL-6. Mean values, standard deviations, and levels of significance (* : *p* < 0.1, ** : *p* < 0.01, *** : *p* < 0.001, **** : *p* < 0.0001, ns : *p* > 0.1) are portrayed on each chart.

Figure 5 encapsulates the expression of the selected biomarkers in each circuit. MCP-1 demonstrates the least divergence between the various setups, apparent also in its insignificant variance (*p* = 0.2027), as measured using one-way ANOVA. On the other hand, MPO shows the lowest *p*-value (1.7024·10^−13^) under one-way ANOVA; however, this is mainly due to the substantial increase in all circuits except for the control system. Apart from the mean values, the charts illustrate the standard deviations and the level of significance. TRX1 manifests reduced disparity between the individual measurements yet presents adequate significance (*p* = 0.0006). MDA and, to a much greater extent, IL-6 come closest to the ideal embodiment of an oxidative/inflammatory response in each system, as per their individual handling, evincing high levels of significance with *p*-values of 5.5962·10^−9^ and 7.0644·10^−7^, respectively.

Finally, no plasma leakage was witnessed throughout the experimental procedure, nor was there any noteworthy volume loss due to sampling, as testified by the constant pressure drop measurements.

## 4. Discussion

Reactive oxygen species contribute to and arise from numerous cellular pathologies, diseases, and aging [37]. Any imbalance in the production or disposal of ROS triggers oxidative stress that acts as a precursor of inflammatory response [38]. Molecular hydrogen has been reported to be surprisingly effective in countering phenomena of oxidative stress and inflammation in a vast range of occasions/disorders [14,39,40]. The notion of investigating the efficacy of hydrogen gas during extracorporeal gas exchange was premised on the excessive systemic inflammatory response associated with ECMO treatments, and the fact that relevant research is scarce.

A cornerstone of this study has been, from the very beginning, the artificial stimulation of inflammation in circulating blood. LPS was the ideal candidate for this purpose owing to its straightforwardness in terms of experimental handling, accessibility, and comparability. Identifying the most suitable concentration to work with was essential prior to any fully fledged in vitro experiment. This was accomplished by measuring the inflammatory response of blood samples from healthy human donors when exposed to diverse LPS concentrations. As Figure 2 demonstrates, the highest IL-6 expression was observed at 250 ng mL^−1^, hence the decision to work further with this LPS concentration.

Maintenance of physiological blood gas values could only be achieved through controlled gas exchange, as Figure 3 signifies. Although a pO_2_ value in excess of 100 mmHg could also be reached in the non-aerated circuits over time, pCO_2_ continually declined far below arterial values in these systems. This gradual gas transfer has been attributed to the material and wall thickness of the collapsible reservoir (<1 mm); the same elements granting flexibility and collapsibility, enable gas diffusion between the circulating blood and the surrounding environment. On a separate note, despite being perfectly constant over time, the pO_2_ values registered by the BGA upon analyzing blood samples from the H_2_-handled circuit were repeatedly found to be lower than anticipated. Gas samples taken upstream of the gas exchanger sustained similar penalties in pO_2_, according to the blood gas analyzer’s report, raising suspicions of erroneous measurements. To illuminate this obscurity, an in-line pO_2_ gas sensor (FDO2, Pyroscience GmbH, Aachen, Germany) was placed at the inlet of the gas exchanger [41]. The values recorded fell within the predicted range of 130–140 mmHg, thus validating the assumption of BGA measurement inaccuracy in the presence of H_2_, whether in blood or in the gas phase. This predicament needs to be further investigated to ascertain the origins of the error and take the necessary measures prior to any in vivo trial.

The concentration of dissolved hydrogen in blood remained relatively constant, well within the expected margin, as Figure 3d denotes, despite the incessant losses towards the environment. In fact, the rate at which hydrogen can escape through most materials, on account of its minute size, renders constant gas supply compulsory. The target concentration of 100–120 ppb is an average of the most frequently implemented concentrations in ongoing pertinent studies [14]. As a side note, the hydrogen monitoring water system (HWMS) fitted exclusively onto the H_2_-supplied circuit systematically yielded consistent results, whose accuracy has been validated through exhaustive trials and sensor recalibrations [41].

Figure 4 underlines the gravity of blood trauma as a critical parameter to be reckoned with when conducting gas exchange investigations. In view of the small scale of the circuits and the duration of the experiment, the magnitude of hemolysis in the uncontaminated (LPS-free) systems is not out of the ordinary, as similar studies attest [42]. The low blood volume and protracted experimental runtime, in association with the lack of any physiological regulation mechanisms during in vitro trials, are presumably the main culprits for the severely higher blood trauma in the LPS-infused circuits. Still, the recorded hemolysis rate did by no means influence the gas exchange capacity of blood, as witnessed in the pertinent charts (Figure 3). Finally, the notable reduction of blood trauma in the presence of hydrogen is an intriguing observation that requires further analysis.

Evidence of the antioxidative and anti-inflammatory action of molecular hydrogen is provided in Figure 5, where the expression of each biomarker in every system is documented. Specifically:

- MCP-1 levels remain practically unchanged throughout the investigation in all the circuits. This may indicate a low cell count that hampers any noticeable observation in MCP-1 expression when blood stems from healthy donors. The mild concentration increase in the LPS systems confirms the low number of cells being triggered.
- The majority of circuits exhibit a tendency towards the concentration in the Control with regard to MPO measurements. The concentration difference between the C and Ref/LPS systems leaves a small margin for H_2_ to act, similar to MCP-1. The slight variance between the different setups points towards time-dependent cell activation rather than any shear rate-induced stress. Nevertheless, these findings might provide a clue concerning in vivo investigations, where cell variability occurs over time, and in vivo activation mechanisms come into effect, potentially allowing for better evaluation of hydrogen’s efficacy.
- TRX1 expression does not seem to be affected by H_2_ as it is with MPO. This agrees with the fact that TRX1 and MPO are associated with anti-inflammatory and pro-inflammatory activity, respectively. Hence, lower MPO concentrations correspond to higher ones for TRX1.
- Varying levels of MDA expression can be witnessed among the systems, suggesting a combination of time-associated and mechanical stress. The inflammatory response to LPS is once again countered by the treatment with H_2_, although not to baseline levels. This agrees with Huang’s findings, where lower MDA concentrations were observed in the hydrogen-treated lungs (as opposed to the nitrogen-treated ones) [17,18].
- IL-6: apparent string inhibition by H_2_. In contrast to MDA, the unambiguously strong IL-6 suppression makes a compelling argument concerning hydrogen’s anti-inflammatory action and the specific pathways being inhibited, as already reported [43]. Furthermore, as with MPO, IL-6 also has pro-inflammatory characteristics, and since it is so heavily suppressed, the anti-inflammatory activity might naturally be reinforced (i.e., TRX1).

Finally, in spite of any disparity in blood gas data between the gas-exchange-handled circuits and the “non-ventilated” ones, there was no trace of deviating biomarker expression in sister systems (e.g., Ref–Ref*).

Some of the above findings raise the issue of the number of blood cells, which directly relates to the overall blood volume of the circuits. This has been a hindrance ever since the decision was made to conduct the investigations with human blood, which required drastic downsizing of all the components. For the same reason, the circuit Ref*H_2_ was omitted from these investigations, especially since hydrogen is not expected to react with healthy blood, as some preliminary measurements have shown. The matter of an inexhaustible blood supply could perhaps be addressed by taking advantage of the hospital’s blood bank reserves (set aside for quality control purposes). Unfortunately, blood bags usually contain citrate phosphate dextrose (CPD) as an anticoagulant. This interfered with the study’s design, which intended heparin as an anticoagulant in pursuit of keeping the results as clinically relevant as possible. 

Ultimately, the experimental output of the study is very positive, corroborating hydrogen’s preventative action and therapeutic potential in ECMO settings and laying the groundwork for future research and subsequent medical applications. A sequel to this study could offer invaluable information by exploring several parameters, such as:

- the impact of blood volume on biomarker expression
- a wider spectrum of biomarkers
- the influence of hydrogen’s concentration on its curative efficacy
- hydrogen’s antioxidant and anti-inflammatory action at different administration patterns (e.g., continuous, intermittent, or delayed)

Moreover, recruiting a substantially larger pool of donors, representing both sexes as well as certain groups of patients, shall bestow upon said study essential gravitas. 

Future studies should also consider the duality of hydrogen’s therapeutic potential. Interestingly, H_2_ in the form of hydrogen sulfide (H_2_S) has been shown to have a significant influence on immune cells. It can modulate the inflammatory response of macrophages, potentially alleviating proinflammatory phenotypes [44]. However, continuous exposure to high levels of H_2_S can lead to immune suppression, triggering oxidative stress, inflammation, apoptosis, and an imbalance in T helper cells [45]. On the other hand, the use of molecular hydrogen has been found to be a protective agent against radiation-induced immune dysfunction, reducing oxidative stress and apoptosis and regulating T-cell balance [46,47]. Moreover, supraphysiological levels of hydrogen peroxide—one of the most common ROS—trigger vasoconstriction, endothelial dysfunction, hypertension, and a proinflammatory state [48]. It is, therefore, possible that hydrogen peroxide may contribute to the inflammatory response during ECMO treatment. The occasionally contradictory outcome of hydrogen administration and the potential dependence on the composition of the carrier compound entreat further research to fully understand the impact of H_2_ on cellular function, which in several cases can only be addressed by long-term in vivo models.

## 5. Conclusions

This study attempts to ascertain the therapeutic potential of molecular hydrogen in extracorporeal gas exchange settings. With this objective in mind, several ECMO circuits have been assembled and tested in vitro using fully heparinized blood from healthy human donors. The findings reveal elevated blood trauma in LPS-infused circuits, whereas gas exchange and the balance of blood gases at physiological levels do not seem to have any particular influence on the inflammatory outcome. On the contrary, H_2_ has proven to be particularly competent in counteracting both oxidative and inflammatory stress, as evinced by diverse biomarkers, notwithstanding its ameliorative action in terms of hemolysis.

## Figures and Tables

**Table 1:** Inventory of the systems tested in this study and their fundamental properties. The star-flagged setups were additionally equipped with a miniature gas exchanger module.

System	Label	Total Volume V_prim_ [mL]	Blood Flow Rate Q_B_ [mL min^−1^]	Gas Mixture Content [%]		
				Air	CO_2_	H_2_
Control	C	1.5	-	-	-	-
Reference 1	Ref	45	40	-	-	-
Reference 2	Ref*	70	40	96	4	-
LPS 1	LPS	45	40	-	-	-
LPS 2	LPS*	70	40	96	4	-
LPS 3	LPS*H_2_	70	40	90	4	6

**Table 2:** List of the investigated biomarkers.

Biomarker	Abbreviation	Classification
Monocyte chemoattractant protein-1	MCP-1/CCL2	oxidative stress/inflammation
Myeloperoxidase	MPO	oxidative stress/inflammation
Thioredoxin-1	TRX1	antioxidant/anti-inflammatory
Malondialdehyde	MDA	oxidative stress
Interleukin 6	IL-6	pro-inflammatory

## Data Availability

The raw data supporting the conclusions of this article will be made available by the authors, without undue reservation.
